# Diverse Views and Practices on the Handling of Explanted Hardware Highlights the Need for Standardized Management

**DOI:** 10.1111/os.14327

**Published:** 2024-12-30

**Authors:** Ali Engin Daştan, Arman Vahabi, Hüseyin Günay, Kemal Aktuğlu

**Affiliations:** ^1^ Department of Orthopedics and Traumatology Ege University School of Medicine Izmir Turkey; ^2^ Department of Hand Surgery Izmir City Hospital Izmir Turkey

**Keywords:** device, implant, removal, removed, survey

## Abstract

**Objective:**

Orthopedic implants may need to be removed for various reasons. There is little data on the appropriate handling of implants after their removal from patients. This study aimed to analyze how orthopedic surgeons handle removed implants and their underlying philosophies, using data collected from a survey.

**Methods:**

This study, conducted between May 2024 and June 2024, utilized an online survey targeting orthopedic surgeons and residents in Turkey to investigate practices and views regarding removed implants. A total of 205 participants completed an 11‐question online survey via Google Forms. The survey covered hospital types, professional experience, protocols for handling removed implants, practices for archiving and disposing of implants, and perspectives on current practices and future direction.

**Results:**

Participants' professional experience varied widely. None of the participants followed a specific protocol for managing removed implants. Opinions on giving implants to patients were diverse: 17.1% would never give the implant to the patient, 32.2% would comply with the patient's request, and 50.7% had no definitive approach. A minority (2.9%) systematically archived implants, while others archived selectively or disposed of them as medical waste. The primary motivations for archiving included medicolegal protection (21%) and professional curiosity (75.2%). Only 2.9% had experience with legal requests for removed implants, and 80% supported establishing regulations for handling removed implants.

**Discussion:**

Orthopedic surgeons' legal and ethical perceptions regarding removed implants, as well as their preference of handling, vary widely. Establishing a standardized approach can reduce this variability in practice and ensure uniformity in healthcare.

## Graphical abstract

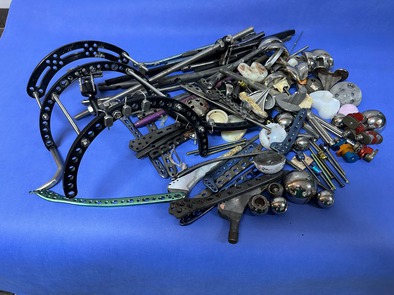

## 1 Introduction

Orthopedic implants may need to be removed for various reasons, including revision surgery, infection, discomfort, nonunion, implant failure, and the removal of implants placed in growing bone [1, 2, 3]. It has been reported that implant removal surgeries constitute approximately 5% of all surgeries performed in orthopedic practice [4, 5]. The literature on orthopedic implant removal primarily centers around studies focused on the indications for removal, the complications encountered during the procedure, and the functional gains achieved postexplantation [3, 6, 7, 8].

In different countries, and even among different states within the same country, various legal frameworks have been proposed regarding the handling of implants after their removal from patients [6, 9, 10, 11]. The three main axes of these discussions are ethical and legal concerns regarding the ownership of the removed implant, if the implant is returned to the patient then what would be the extent of responsibility for secondary complications such as infection and injury that may be caused by the removed implants, and how these implants, once recorded in national registry databases during implantation, should be managed postremoval. However, setting aside the debate over ownership of the implant, there is minimal discussion regarding the optimal handling of removed implants [11, 12].

To our knowledge, there are no established guidelines for orthopedic surgeons to follow regarding the handling of removed orthopedic implants. Factors such as the policies of the hospital they work in, stance of clinic chief, the type of implant removed, the surgeon's personal approach, and surgeons' previous legal precedents all contribute to the variability in how this hardware are managed. The purpose of this study was to evaluate the practices and perspectives of orthopedic surgeons on explanted hardware, specifically: (i) to evaluate practices and perspectives of handling and of explanted hardware, (ii) to evaluate current regulations or legal considerations governing the management of explanted hardware, and (iii) to evaluate perspectives on future directions for improving the management of explanted hardware.

## 2 Materials and Methods

This study was designed as an online survey targeting orthopedic surgeons and residents in Turkey. Responses to the survey were collected between May 2024 and June 2024. Ethical approval was obtained from the local ethics committee of Ege University under code 24–4.1 T/24. An online survey was prepared using Google Forms. To ensure anonymity, no email verification or name was required for participation in the survey.

### 2.1 Survey Participants

Invitation to participate in the survey was sent through an actively used email group comprised Turkish orthopedic surgeons and residents, where community announcements are regularly shared. Invitation was repeated after one week. The study design did not address the indications for orthopedic implant removal, the timing of removal, or the expected outcomes. The survey consisted of 11 questions aimed at investigating variety of the actions taken by participating orthopedic surgeons regarding removed implants and the philosophy behind their decisions. The survey was conducted in Turkish and took approximately 3 min to complete. Inclusion criteria for the study required participants to be either orthopedics and traumatology specialists or residents and to have answered all questions in the survey. Exclusion criteria were based on inconsistencies in the responses. From the 207 individuals who completed the survey, the results of 2 participants with inconsistent answers were excluded. Data from the total of 205 participants included into final analyses.

### 2.2 Structure of Survey

The first two questions inquired about the level of the hospitals where the participants worked and their professional experience in orthopedics. Corresponding with the organization of the healthcare system in Turkey, the possible responses to the question regarding the hospital type included: second‐level state hospital, third‐level research hospital, third‐level university hospital, private hospital, and private practice. Responses to the professional experience question were categorized into the following ranges: 1–5, 6–10, 11–15, 16–25, and over 25 years. The next two questions asked participants whether they were aware of any existing protocol for handling explanted implants and, if so, to specify that protocol.

In the fifth question, participants were asked about their preference for giving the explanted implant to the patient if requested by the patient. The sixth question inquired about their general practices regarding the disposition of removed implants. For those who indicated that they archived the removed implants, the seventh, eighth, and ninth questions asked which types of implants they primarily archived, the purpose for archiving these implants, and where they stored these archives. Unlike the other questions in the survey, which allowed only a single response, these questions permitted participants to select multiple answers (Table 1).

**TABLE 1:** Questions and possible answers of applied survey.

Question	Answer	Selection
1. What type of hospital are you currently working?	Second‐level state hospital	Only one selection is allowed
	Third‐level research hospital	
	Third‐level university hospital	
	Private hospital	
	Private practice	
2. What is your total experience in the field of orthopedics?	1/2/3/4/5/6/7/8/9/10/11/12/13/14/15/16/17/18/19/20/21/22/23/24/25+	Selection between any year from 1 to 25+ years, only one selection is allowed
3. Do you follow any guidelines, regulations, or protocols regarding the handling of removed implants?	Yes	Only one selection is allowed, If the answer is yes, then next question is activated
	No	
4. Which specific protocol do you adhere to?	Open‐ended question	This question was an open‐ended one
5. If a patient requests to collect the removed implant, do you return the implant to them?	I do not have a standard approach, in some cases, I give the implant to the patient depending on the type of implant.	Only one selection is allowed
	I definitely do not return it in any case.	
	I definitely provide the implant to the patient if they request it	
6. What is your standard procedure for management of explanted implants?	I include it in the medical waste disposal process	Only one selection is allowed
	I archive nearly all of the implants I remove by recording the patient's information	
	I archive almost all of the implants I remove, but I do not record patient information	
	I only archive implants that I find interesting and those that are not commonly encountered in routine practice	
	I only archive implants in cases where I believe they may be involved in a future judicial or medicolegal process	
	I do not archive any implants I remove, and I do not have a standardized approach that I follow	
	If the patient requests it, I provide the implant to them; if they do not want it, I include it in the medical waste disposal process	
7. Which types of implants do you most commonly archive?	Frequently used, currently available trauma implants	Multiple selection is allowed
	Trauma implants that are not frequently used in current practice	
	Frequently used, currently available arthroplasty implants	
	Arthroplasty implants that are not frequently used in current practice	
	I archive implants that are no longer manufactured or are unavailable	
8. If you archive the implants you remove, what is the purpose of doing so?	I archive them for documentation purposes, anticipating potential future judicial or medicolegal proceedings	Multiple selection is allowed
	I archive them out of personal curiosity	
	I archive them to prevent misuse by third parties (e.g., use in another patient)	
9. If you archive the implants you remove, where do you store this archive?	At home	Multiple selection is allowed
	At workplace	
10. Have you ever experienced a situation where an implant you removed was requested by you for a judicial or medicolegal process?	Yes	Only one selection is allowed
	No	
11. Do you think there should be regulations regarding the handling of removed implants?	Yes	Only one selection is allowed
	No	

In the tenth question, participants were asked whether they had ever been requested to provide a previously explanted implant due to a judicial or medicolegal process. In the final question, participants were asked whether they believed that regulations regarding the handling of removed implants were necessary.

### 2.3 Statistical Analyses

The survey data were analyzed using IBM SPSS Statistics version 26. Responses were expressed as frequencies and percentages. Independent variables were evaluated as cross‐sections, and differences in distribution were assessed using the Pearson chi‐square test. A p‐value of less than 0.05 was considered statistically significant.

## 3 Results

### 3.1 Participants

Regarding the type of employed institution, the distribution of participants was as follows: 61 participants were employed at the third‐level university hospitals, 72 at the third‐level research hospitals, 35 at second‐level state hospitals, 28 at private hospitals, and 9 in private practice. Regarding professional experience in orthopedics, 63 participants had 1–5 years of experience, 65 had 6–10 years, 35 had 11–15 years, 11 had 16–20 years, 11 had 21–25 years, and 20 participants had more than 25 years of experience.

### 3.2 Handling of Explanted Hardware

Although 2 participants stated that they followed a protocol for the management of removed implants, they were unable to refer to a specific valid protocol. Regarding opinions on giving the removed implant to the patient, 35 participants (17.1%) stated that they would never give the implant to the patient, even if requested. In contrast, 66 participants (32.2%) stated that they would definitely comply with the patient's request. The remaining 104 participants (50.7%) indicated that they did not have a definitive approach and that their practices varied on a case‐by‐case basis.

### 3.3 The Archiving of Explanted Hardware and the Underlying Motivations for Archiving

Responses regarding the general preferences for handling removed implants revealed that 13 participants (6.3%) archived nearly all the implants they removed, albeit in a nonsystematic manner. Six participants (2.9%) reported systematically archiving almost all removed implants, noting patient information. Twenty‐eight participants (13.7%) indicated that they only archived implants they believed might be subject to future medicolegal processes. Fifty‐three participants (25.9%) archived implants they found interesting or that piqued their professional curiosity. Seventy‐three participants (35.6%) stated that if the patient requested the implant, they provided it to the patient; otherwise, they included it in the medical waste disposal process. Thirty‐eight participants (18.5%) mentioned that their general approach was to include the implant in the medical waste process. Finally, 34 participants (16.6%) reported having a variable approach.

**FIGURE 1:**
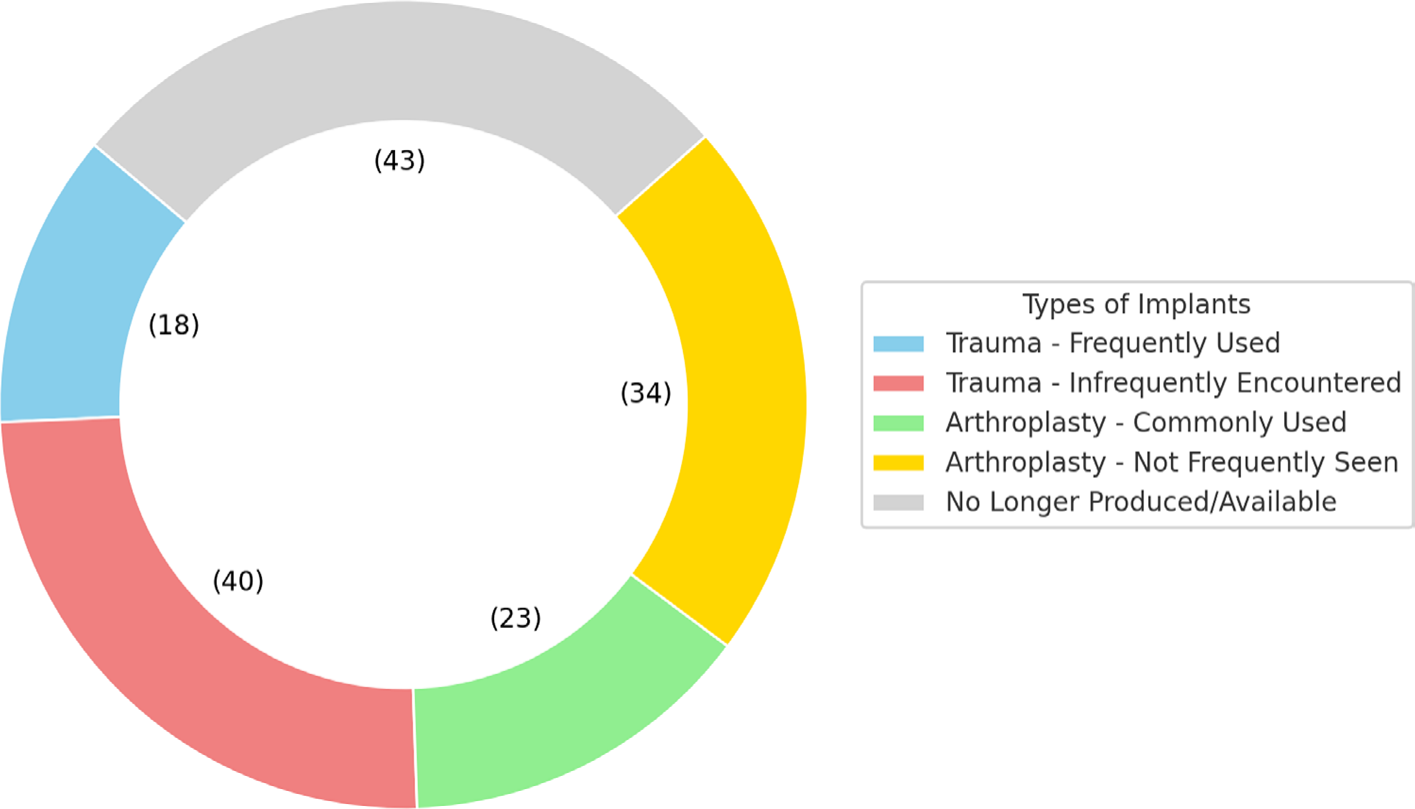
Types of archived implants.

An analysis of responses from the 105 participants who reported archiving some or all the implants they removed revealed diverse practices. Nineteen participants indicated they archive trauma implants frequently used in current routine practice, while 40 participants reported archiving trauma implants, which they believe are infrequently encountered in routine practice. Additionally, 23 participants stated they archive arthroplasty implants commonly used in their practice. Thirty‐five participants preferred archiving arthroplasty implants that are not frequently seen in routine practice, and 44 participants reported archiving implants that are no longer produced or available in Turkey (Figure 1). In response to the question about where they kept their archive, 66 participants stated that they stored the implants at their workplace, while 37 participants reported keeping them at home.

**FIGURE 2:**
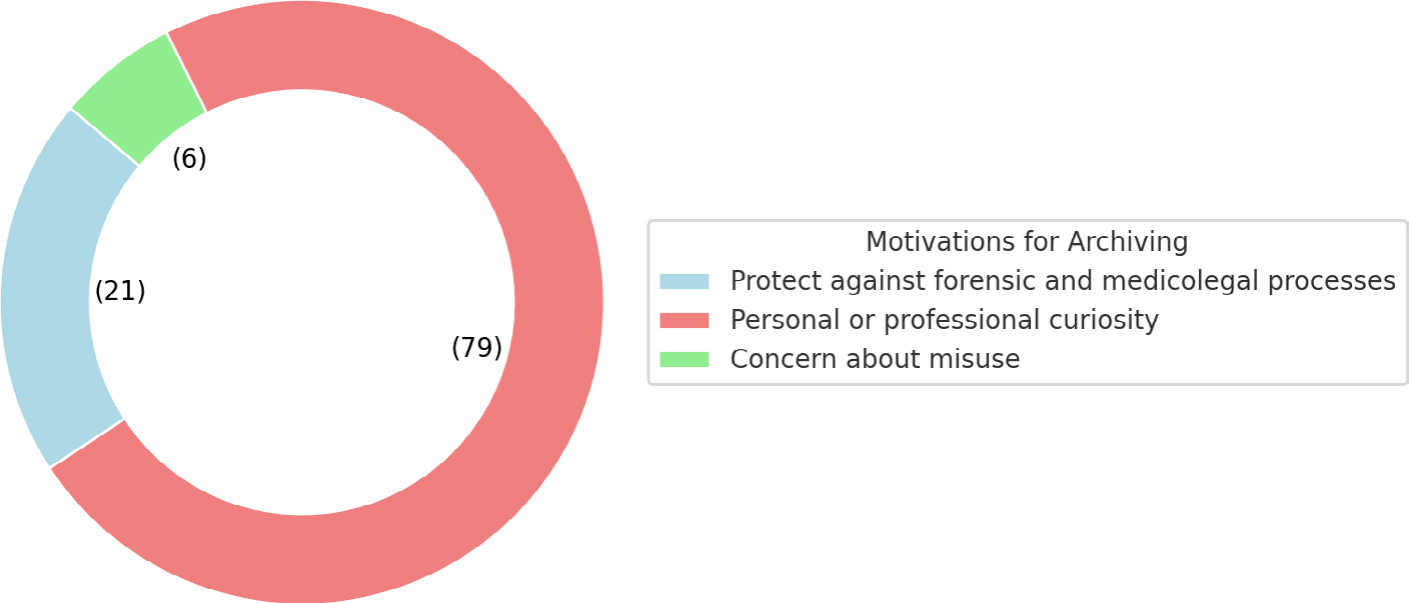
Motivation for archiving implants.

Regarding the motivations for archiving among the 105 participants who reported archiving some or all the implants they removed, 22 (21%) participants indicated they archived implants to protect themselves against potential future forensic and medicolegal processes. Seventy‐nine participants (75.2%) stated that their archiving was driven by personal or professional curiosity. Additionally, seven participants (6.6%) expressed concern that the implants might be misused if not archived, which motivated their decision to archive them (Figure 2).

### 3.4 Regulations on Explanted Hardware

Six participants (2.9%) reported having been requested to provide a removed implant as part of a judicial or legal process. Furthermore, 164 participants (80%) indicated that they believe establishing regulations for the handling of removed implants is necessary. This opinion was formed independently of the type of institution (*χ* ^2^ = 8.343, *p* = 0.138) and the participant's experience in orthopedics (*χ* ^2^ = 2.029, *p* = 0.730).

## 4 Discussion

The main finding of this study was the considerable variation among orthopedic surgeons in how removed implants were handled and the underlying philosophies guiding these practices. The absence of established guidelines for surgeons on this issue appears to be a fundamental reason for this diversity.

### 4.1 Handling and Archiving of Explanted Hardware

Although implant removal constitutes a significant part of orthopedic practice, there is very little literature on the subject, particularly regarding the handling of removed implants. The current literature seems to focus more on discussion about legal and ethical aspects of returning the implants to the patient [11, 12, 13, 14]. However, this issue is far more complex and multifaceted. Although there are limited data available to analyze the legal frameworks in different countries and their practical applications, some argues that removed implant should be considered part of the patient, thus should be returned to patient if asked. But we think this approach raises challenging questions about its general applicability. Considering 17% of respondents stated they would not return the implant to the patient under any circumstances, we can speculate that this perspective is not universally accepted. In the next step, even more complex questions arise: If we accept that the ownership of the implant belongs to the patient, what should be the optimal handling of the implant if the patient does not wish to receive it? Conversely, if we accept that the implant's ownership does not belong to the patient and should not be given to them, what protocols should be followed for removed implants? Should the patient have a say in how the implant is managed even if it is not given to them? Should this approach vary depending on the type of implant? Is it allowed for the surgeon to keep it? Should these implants be archived systematically, if so, is it the surgeon's responsibility to undertake this archiving? Considering that 38.5% of the participants archiving removed implants in some manner, it is essential to evaluate the ethical and legal suitability of archiving from a multifaceted perspective, which is hardly ever argued in the previous literature.

### 4.2 Regulations and Legal Considerations on Explanted Hardware

National implant registry databases are another crucial aspect of implant‐related regulations. Arthroplasty registration systems have been in place for a long time in many countries. As part of the new European Union Medical Device Regulation, which is planned to be completely taken into action within a few years, all implants are planned to be recorded in a database with efforts to improve safety and quality measures [10, 15]. Considering that one of the purposes of registry systems is to access data regarding outcomes of the implants used, and this scope is intended to be expanded to all implant types, allowing patients to retain removed implants or leaving their management to the physician's discretion seems to contradict the objectives of these systems. Although in some instances analyzing the explanted implants to detect potential issues with implants becomes necessary (like in cases of retrieved implants), the necessity of a general regulation covering this situation, which affects a relatively small group, is debatable [15, 16]. However, the fact that 5% of the participants archive implants they believe may be subject to future judicial proceedings indicates that these discussions are relevant to surgeons' daily practices and should be discussed further.

### 4.3 Future Direction for Handling of Explanted Hardware

The idea of including removed implants in reuse or recycling processes has been proposed in various studies [9, 17, 18, 19]. While there are few studies on this subject, questions remain about the economic viability of such efforts [20]. However, it is important to consider this issue from both environmental and economic perspectives. Arguments against the reuse of removed implants include the potential risk of infection, structural changes in the implant and related complications [21]. However, thinking that daily surgical instruments can be reused indefinitely after undergoing sterilization processes, and in many underdeveloped and developing countries, products marketed as disposable by the manufacturer are reused multiple times, it does not seem fair to reject this opinion at once [20, 22]. Although the permanent placement of implants in a patient's body differentiates these examples, it is unclear where to draw the threshold for reuse suitability for different types of implants. In their study on the systematic recording and reuse of removed implants, Danesi et al. reported a high suitability rate of 66% for reuse of trauma implants [22]. Additionally, a study from Sweden reporting results of national regulation that took place in 2015, indicating that approximately $250 million is saved annually from gathering and recycling implants after cremation [9]. Related to this debate, 3.4% of participants expressed concern regarding possible misuse of explanted implants. They stated possibility of misuse of removed implants by third parties without their approval was their motivation for archiving such implants. We posit that this perspective, while seemingly minor warrants further consideration. We believe that establishing protocols for recycling or reusing removed implants, in line with sustainability and environmentally friendly policies, is a necessary step that is long overdue. On the other hand, these discussions should also include additional costs that would be incurred if removed implants, were recorded, archived, or processed.

The management of removed implants will inevitably be shaped by institutional and national regulations, as is the case with many healthcare services. However, it is our duty to develop the scientific groundwork that will guide these regulations. As noted by 80% of the participants, there is a significant need for comprehensive guidelines to address aspects not explicitly delineated by current legal frameworks.

### 4.4 Limitations and Strengths

The primary limitation of our study is that the survey was conducted exclusively in one country. While this focus may increase the likelihood that legal and ethical dilemmas are influenced by national variables, potentially causing information bias to draw global conclusions, the absence of an accessible legal framework and relevant medical literature in many countries suggests that the issue addressed by this study is a global problem. Future multicenter, multinational studies could uncover different dimensions of the problem and pave the way for the development of a more comprehensive approach.

## 5 Conclusion

Orthopedic surgeons' legal and ethical perceptions regarding removed implants, as well as their preference of handling, vary widely. Establishing a standardized approach can reduce this variability in practice and ensure uniformity in healthcare.

## Author Contributions

*Design*: A.V., K.A., and H.G.; *Data Acquisition*: A.V. and A.E.D.; *Writing*: A.V. and A.E.D.; *Critical Review*: H.G. and K.A. All authors read and approved the final manuscript.

## Ethics Statement

Ethical board approval was obtained from the Ege University Local Ethics Committee (24–4.1 T/24).

## Conflicts of Interest

The authors declare no conflicts of interest.

## Data Availability

The dataset for that study is available upon reasonable request from the corresponding author. Graphical figures are created using artificial intelligence.
